# Novel Framework for Simulated Moving Bed Reactor Optimization Based on Deep Neural Network Models and Metaheuristic Optimizers: An Approach with Optimality Guarantee

**DOI:** 10.1021/acsomega.2c06737

**Published:** 2023-02-07

**Authors:** Vinícius V. Santana, Márcio A. F. Martins, José M. Loureiro, Ana M. Ribeiro, Luana P. Queiroz, Carine M. Rebello, Alírio E. Rodrigues, Idelfonso B. R. Nogueira

**Affiliations:** † Laboratory of Separation and Reaction Engineering, Associate Laboratory LSRE-LCM, Department of Chemical Engineering, Faculty of Engineering, University of Porto, Rua Dr. Roberto Frias, 4200-465 Porto, Portugal; ‡ Industrial Engineering Program, Polytechnic School, Federal University of Bahia, Rua Prof. Aristides Novis, 2—Federação, 40210-630 Salvador/Bahia, Brazil; § Department of Chemical Engineering, Norwegian University of Science and Technology (NTNU), 7491 Trondheim, Norway

## Abstract

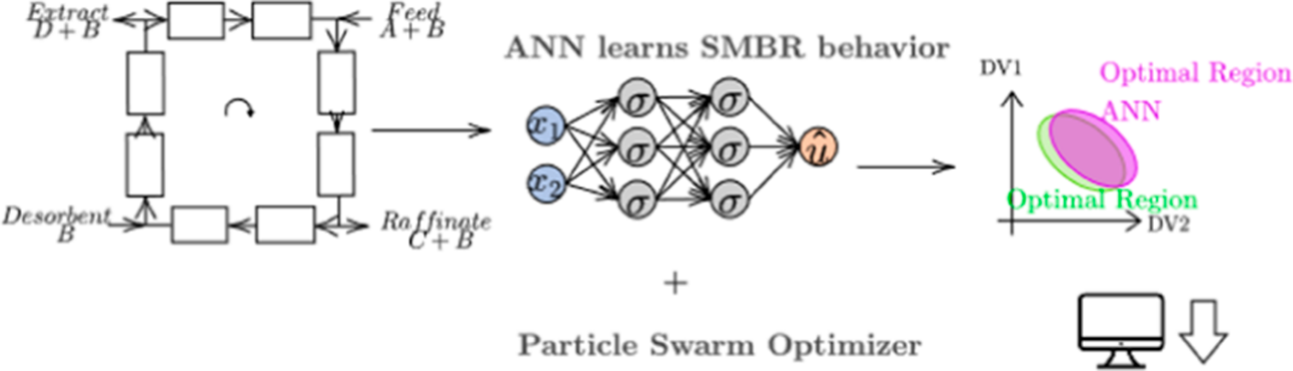

Model-based optimization of simulated moving bed reactors (SMBRs) requires efficient solvers and significant computational power. Over the past years, surrogate models have been considered for such computationally demanding optimization problems. In this sense, artificial neural networks—ANNs—have found applications for modeling the simulated moving bed (SMB) unit but not yet been reported for the reactive SMB (SMBR). Despite ANNs’ high accuracy, it is essential to assess its capacity to represent the optimization landscape well. However, a consistent method for optimality assessment using surrogate models is still an open issue in the literature. As such, two main contributions can be highlighted: the SMBR optimization based on deep recurrent neural networks (DRNNs) and the characterization of the feasible operation region. This is done by recycling the data points from a metaheuristic technique—optimality assessment. The results demonstrate that the DRNN-based optimization can address such complex optimization while meeting optimality.

## 1 Introduction

The simulated moving bed reactor (SMBR) extends the simulated moving bed (SMB) process, where the continuous chromatographic separation enhances chemical reactions. The SMBR has attracted significant attention in the past years,^1,2^ especially for synthesizing oxygenated compounds and isomerization reactions.^3,4^

Model-based optimization of SMBR units implies the simultaneous solution of dynamic nonlinear partial differential equations, requiring efficient solvers and significant computational power. Moreover, several other issues arise in optimizing SMBRs due to the coupling of reaction and separation. It reduces the degree of freedom;^5^ that is, fewer variables can be manipulated to achieve purity and conversion requirements and conflicting objectives; namely, improving one performance indicator worsens the others in a nontrivial way.

Therefore, SMBR optimization is not a trivial task that motivated many studies to address it with mixed approaches—first-principles models with varying simplifications and deterministic/heuristic optimization methods. Tie et al.^6^ proposed an epsilon-constrained multiobjective optimization with a full-discretization model to maximize the production rate of propylene glycol methyl ether acetate and conversion of ethyl acetate solved with interior point OPTimization techniques. Ray and Ray^7^ proposed a multiobjective optimization using nonsorted genetic algorithm II (NSGA II) to maximize yield and purity in biodiesel production. Subramani et al.^8^ presented a multiobjective optimization problem for the methyl tertiary butyl ether synthesis in a Varicol SMBR optimized with the NSGA algorithm. Nogueira et al.^9^ proposed a single objective optimization problem for producing *n*-propyl-propionate with true moving bed reactor approximation using a particle swarm optimization (PSO) method.

The optimizer solves the SMBR model several times to evaluate the objective function during optimization. The simulation time can grow significantly depending on model simplifications—reaction rate law, equilibrium equations, number of columns per zone, and nonisothermal operation. For the design of the SMBR, the optimization time may not be prohibitively high. However, the high-fidelity model may become an unfeasible option in an actual plant coupled with up- and downstream equipment or in real-time applications. Simplifications of the high-fidelity model are often employed for SMB separation by using the true moving bed (TMB) concept and the SMBR with the true moving bed reactor (TMBR). However, it has been shown that this approximation can fail to describe the SMB and SMBR in several scenarios.^10,11^

Over the past years, reduced-order/surrogate models have been considered for optimization problems. Surrogate models are mathematical models identified using statistical techniques and attenuating the associated computational costs of an optimization problem. In this sense, artificial neural networks—ANNs—have found applications for these systems for modeling the SMB unit (without reaction).^12−14^ However, they have not yet been reported for the reactive SMB (SMBR). ANNs are powerful models composed of multiple processing layers that learn representations of data with various levels of abstraction^15^ and are proven to be able to approximate any nonlinear *C*_1_ continuous functions^16^ and overcome the curse of dimensionality.

Despite ANNs’ high accuracy for nonlinear function approximation, assessing their converges and capacity to represent the optimization landscape competently is essential.^17^ To this end, it is important to use strategies to identify high-accuracy surrogates (low bias) and investigate the optimality, that is, convergence to the true global optimum. However, a consistent method for optimality assessment using surrogate models is still an open issue in the literature. Optimality is usually assessed by visual inspection and point-wise (i.e., by comparing the high-fidelity model-based optimal point with the surrogate model-based optimal one).

A few works have recently shown one approach to evaluating the optimization results with a cluster of points instead of a single evaluation. Park (2013) proposed a bootstrap approach, and Nogueira et al.^18^ and Rebello et al.^19^ used recycled data from PSO—however, using the high-fidelity model. As shown in refs (9) and (19), it is possible to map the optimal region through the data population generated from the PSO algorithm. As such, the method allows using an available massive database from PSO to produce meaningful information about the optimal solution and increase its robustness level by evaluating its associated uncertainty. Addressing this issue is one main contribution of this work and still an open issue in the literature.

Hence, two main contributions of this work can be highlighted: the SMBR optimization based on deep neural networks (DNNs) and the challenging characterization of the feasible operation region (FOR). Therefore, the DRNN-based optimization is assessed in the optimization of an SMBR unit. This is done by recycling PSO data points, allowing the optimality assessment. A case study presents the synthesis of *n*-propyl-propionate in an SMBR-4 unit. The present work proposes a novel framework for SMBR units’ PSO-oriented optimization based on DNN models.

## 2 Methodology

### 2.1 SMBR Model

**Figure 1:**
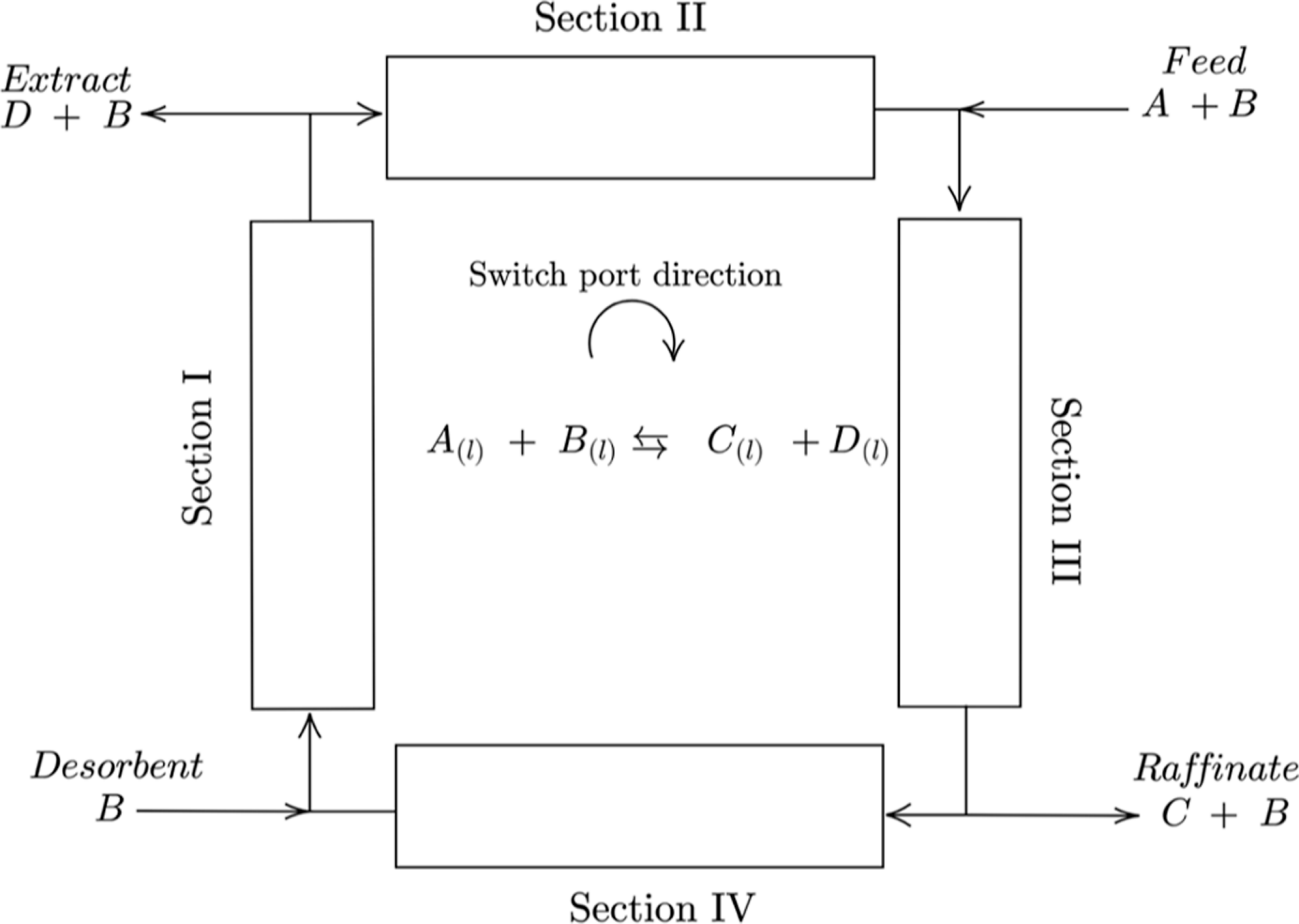
Diagram of a SMBR unit.

The SMBR comprises a set of fixed-bed columns arranged in a recirculation loop. There are two inlets (feed and desorbent) and outlet streams (extract and raffinate). The packing material(s) have either adsorptive or catalytic properties, and the feed stream contains the reactant(s). The products are separated from the reactants as the reaction occurs within the unit. Figure 1 illustrates a four-zone SMBR for a reversible bimolecular hypothetical reaction. For such cases, it is usual that one of the reactants is used as a desorbent. The reactants (A + B) are fed between sections II and III; the raffinate stream is located between sections III and IV. It is where the less adsorbed product (C) is collected, the desorbent stream is located between sections I and IV, and the extract stream lies between sections I and II from which the more adsorbed product (D) is withdrawn.

**Figure 2:**
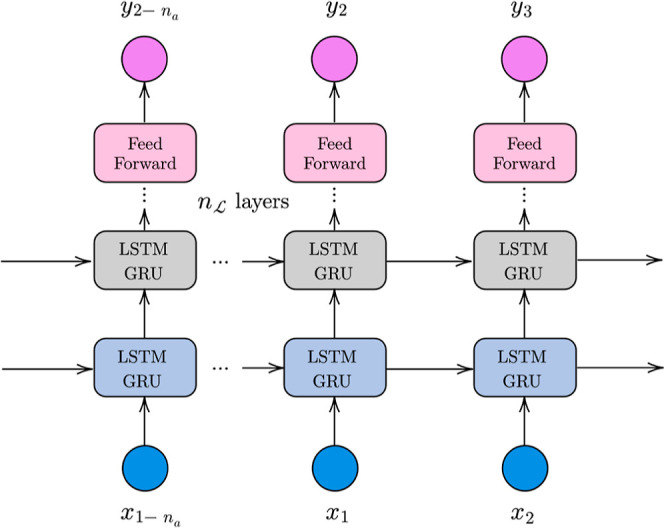
Proposed stacked RNN model.

In the present work, the SMBR-4 model for the synthesis of *n*-propyl propionate proposed in ref (11) was implemented in gPROMs^20^ to serve as the high-fidelity model to be optimized and source of data for the DRNN. In this case, propanoic acid, propanol, *n*-propyl propionate, and water play the A, B, C, and D roles, respectively, considering Figure 1 as a reference. The model equations and parameters are displayed in Tables A1 and A2. The parameters were previously estimated by Nogueira et al.^9^

**Table 1:** Input and Output Signals’ Ranges

variable	minimum	maximum
Input Signals (Designed)		
*C*_F_^ProAc^/(mol/L)	1.40 × 10^–1^	1.33 × 10^1^
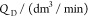	1.00 × 10^–3^	4.99 × 10^–3^
*t*_s_/(min)	1.00 × 10^1^	5.99 × 10^1^
Output Signals		
raffinate purity/(%)	0.65	99.99
conversion/(%)	1.28	99.97
productivity/(mol/L/day)	3.432 × 10^–5^	16.74
desorbent consumption (L/mol)	4.785 × 10^–1^	89.95

**Table 2:** Hyperparameter Search Space for the RNN

hyperparameters	search space
initial learning rate	{1 × 10^–4^, 3.162 × 10^–4^, 1 × 10^–3^}
batch size	{4, 8, 16}
number of recurrent layers	{1, 2, 3}
recurrent layer type	{GRU, LSTM}
number of neurons in the recurrent layers	{100, 180}
activation function in the recurrent layers	{relu, tanh}
number of neurons in the intermediate fully connected layer	{20, 60}
activation function in the fully connected layer	{relu, tanh}

**Table 3:** Results of Best Hyperparameters for Each Performance Indicator for the RNN

hyperparameters	productivity	desorbent consumption	purity	conversion
initial learning rate	1 × 10^–3^	3.162 × 10^–4^	1 × 10^–3^	1 × 10^–3^
batch size	4	4	4	4
number of recurrent layers	3	2	3	3
recurrent layer type	{layer 1: LSTM, layer 2: LSTM, layer 3: LSTM}	{layer 1: GRU, layer 2: GRU}	{layer 1: GRU, layer 2: GRU, layer 3: GRU}	{layer 1: LSTM, layer 2: GRU, layer 3: GRU}
number of neurons in the recurrent layers	{layer 1: 180, layer 2: 180, layer 3: 180}	{layer 1: 180, layer 2: 100}	{layer 1: 100, layer 2: 100, layer 3: 180}	{layer 1: 180, layer 2: 100, layer 3: 180}
activation function in the recurrent layers	{layer 1: relu, layer 2: relu, layer 3: relu}	{layer 1: relu, layer 2: relu}	{layer 1: relu, layer 2: tanh, layer 3: relu}	{layer 1: relu, layer 2: relu, layer 3: tanh}
number of neurons in the intermediate fully connected layer	20	60	60	20
activation function in the intermediate fully connected layer	Relu	relu	relu	Tanh

**Table 4:** Rescaled Decision Vectors at the Optimal Point for Rigorous and DRNN Models (Rescaled to the Original Units of Measurements)

model	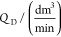	*C*_F_^ProAc^/(mol/L)	*t*_s_/(min)	objective function
DRNN	1.68 × 10^–3^	13.33	47.76	–1.17
rigorous	1.77 × 10^–3^	13.33	47.51	–1.14

**Table 5:** Performance Indicators at the Optimal Point for the Rigorous Model and RNNs

model	purity/(%)	conversion/(%)	desorbent consumption/(L/mol)	productivity/(mol/L/day)
DRNN	99.58	93.10	1.18	3.56
rigorous	97.32	90.86	1.11	3.69

A total of four performance indicators of separation and reaction were considered: raffinate purity, limiting reactant conversion, productivity, and desorbent consumption. Table A1 shows the equations for raffinate purity, conversion, desorbent consumption, and productivity in the case study of propyl-propionate synthesis.

### 2.2 DNN Model—Building and Estimation

Tables A1 and A2 show that the SMBR is an inherently dynamic process. Hence, using the discrete-time recurrent neural network (RNN) model is the most appropriate way of introducing inductive bias and shortening the amount of training data needed for long-horizon predictions (simulation mode). The difference between prediction and simulation is well discussed elsewhere.^14^ Thus, this architecture is proposed as a surrogate model of SMBR-4. Several RNNs, one for each performance indicator (time-averaged raffinate purity, conversion, desorbent consumption, and productivity), were identified, that is, multiple input single output (MISO). Here, we propose a flexible approach with a mixed stack of state-of-the-art RNN architectures built—long–short-term memory (LSTM) or gated recurrent unit (GRU) and feed forward layers. Figure 2 illustrates the proposed approach.

**Figure 3:**
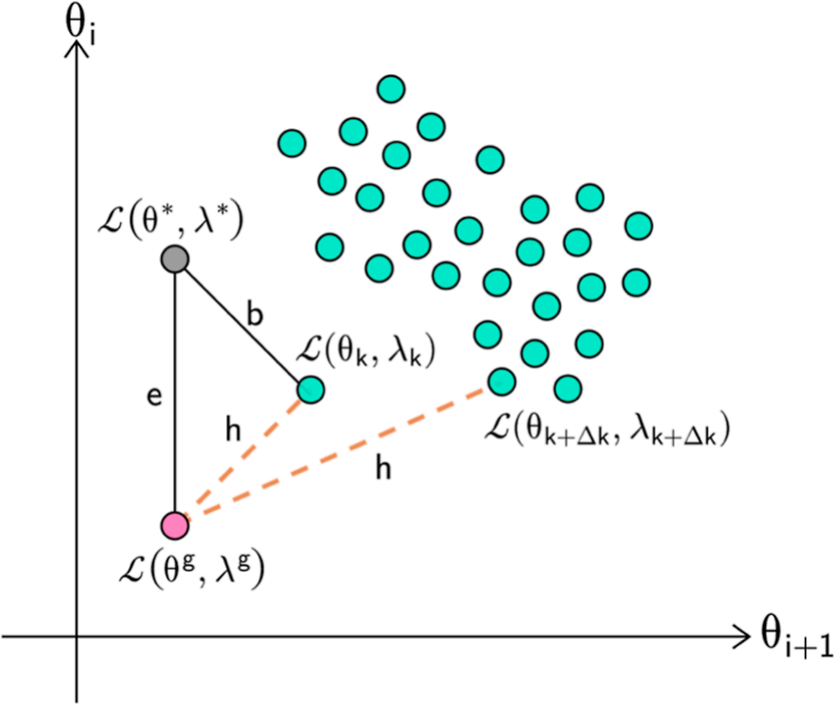
2D geometrical illustration for confidence region derivation.

Once the general model architecture is proposed, the identification procedure, that is, a parameter estimation strategy, has to be developed, which includes the design of experiments (DoE) or data acquisition, time series preprocessing, and the parameter estimation itself, which involves defining the neural network topology, cost function and training policy—training set size, optimizer type, and parameters.

Among all possible input variables in the SMBR, the ones with the highest impact on its performance indicators were chosen, according to a strategy developed by Santana et al.^11^—the concentration of the limiting reactant in the feed stream (propanoic acid), the desorbent flow rate, and the switching time. Then, the DoE was performed using the Latin Hypercube Sampling (LHS) algorithm^21^ to sample the input space () with minimal cross-correlation between variables. The sampled inputs were fed to the high-fidelity model simulator as a sequence of step signals persisting until the system reached the cyclic steady state. The simulator outputs (performance indicators) were collected and stored. The LHS is one among many options available for DoE, which includes the popular Sobol sequences.^22^ Each method has its own advantages and disadvantages. The literature in ANN-based surrogates presents relevant usages of both methods.^19,23−28^

The available time series preparation into “experiments”, often named “time windows”, forces the model to learn the whole response to a step change in the input variables. In simple terms, a set of small chunks of time series are created, wherein each experiment (chunk) *E*_i_ consists of a set of tuples .

The time series preprocessing involves identifying long-term dependencies (order) in the time series data. In the present work, the dynamic system is modeled as a nonlinear state space representation as described in ref (29) It implies that for a given discrete-time dynamic system with white noise *v*(*k*), noisy observations y depend on exogenous inputs *u* and past estimated states *z*. It can be written
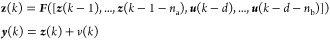
where *n*_a_ and *n*_b_ are the number of past values and *d* is the delay. The system order (*n*_a_ and *n*_b_) is independent of the chosen function used to approximate the true unknown *F* and should be carried before any parameter estimation.^14,30^ To identify *n*_a_ and *n*_b_, the Lipschitz coefficient^31^ method is used.

The neural network topology, cost function, and training policy are known as hyperparameters. They define the optimization problem and must be determined beforehand, that is, before the RNNs’ weights and biases are estimated. The hyperparameter space comprises a set of both discrete and continuous variables, which makes their selection a complex task. In this sense, the state-of-the-art HYPERBAND^32^ optimization algorithm is used here as it wisely allocates resources for training the most promising configurations. The search space for the hyperband comprised continuous and discrete variables: type of each recurrent layer, number of stacked layers, number of neurons per layer, activation function type, learning rate, and minibatch size. Despite using hyperband, other efficient automatic hyperparameter selection methods exist, such as multiobjective optimization problem training loop^33−35^ and TRANSFORM.^36^ At each run of HYPERBAND, the RNNs were trained with least-squares loss function and adaptive moment estimation (Adam) with TensorFlow default parameterization. After selecting the best configuration, the final architecture was trained longer with early stopping. Neural network building, training process, and hyperparameter tuning were implemented in TensorFlow 2.5.

### 2.3 Optimization Problem Formulation

The optimization problem is framed as a single objective function by weighting the SMBR performance indicators constrained by the model equations and decision variables’ limits.

The objective function, evaluated at a cyclic steady state, and constraints can be written as
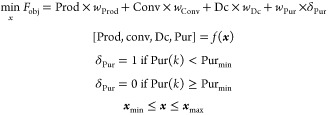
1where Prod, Conv, Dc, and Pur are time-averaged productivity, conversion, desorbent consumption, and purity, respectively, and *x* is the decision vector that contains the input variables values. *w*_i_ are real values that weight each performance indicator; δ_pur_ is an indicator function that penalizes the objective function with *w*_pur_ if the purity is lower than Pur_min_. *f*(*x*) is the model (mechanistic or DRNN) that maps the decision vector to the performance indicators. It can be either the rigorous SMBR-4 model or the RNN.

As shown in the previous section, the SMBR has four main performance indicators usually taken into account: productivity, conversion, purity of the product of interest, and desorbent consumption. The first three are usually desired to be as high as possible and the last as low as possible. It means that the corresponding weights in the objective function should reflect it; that is, the performance indicators to be maximized need a negative weight and vice versa. An in-house implementation of the global PSO method^37^ is used to solve this optimization problem.

PSO was used as the optimization method as it has been demonstrated as an efficient and suitable approach for characterizing confidence regions in optimization problems.^19,38−40^

### 2.4 Feasible Operating Region around the Optimal Point

The strategy for determining the feasible operating region around the optimal point here used was proposed by Nogueira et al.^18^ The uncertainty map is the denominated feasible operating region (FOR) in the referred work. The FOR is a subdomain of process operating variables where the process can attain a defined performance with a certain confidence. Given the population-based optimization method (PSO), a statistical evaluation of the swarm history in the optimization is performed. Usually, the likelihood approach is employed as it considers the system’s nonlinearities. This approach can be employed using the Fisher–Snedecor criterion. All details of the deduction of this criterion for single objective process optimization are beyond the scope of this work. It is presented elsewhere.^19^ Here, the intuition and some mathematical basis is presented.

The key idea is that the Lagrangian (*L*), which is the sum of the objective function with weighted vector of constraints (*C*), can be treated as a random variable, and the decision vector points are statistically compared using the Fisher–Snedecor. The Lagrangian and Fisher–Snedecor can be expressed, respectively, as
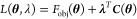
2
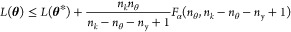
3it compares the Lagrangian  at the minimum found in the optimization, θ*, with a given decision vector θ with objective function *L*(θ) using the Fisher–Snedecor distribution *F*_α_ with confidence level α and *n*_*k*_ – *n*_θ_ – *n*_y_ + 1 degrees of freedom, where *n*_y_ is the total number of performance indicators, *n*_k_ is the number of iterations in the optimization, and *n*_θ_ is the number of operating variables (decision vector). Thus, the history of swarm position and objective function can be evaluated to compose an α confidence-level region when passing the inequality criteria described in inequality (3).

Equation 3 derivation is adapted from the confidence region evaluation for parameter estimation presented by Schwaab et al. (2008)^38^ and Benyahia et al. (2013).^41^ In the proposal of Nogueira et al. (2019)^39^ and here adapted, the methodology was adapted in order to evaluate the feasible operating region of the process operating variables after the optimization.

**Figure 4:**
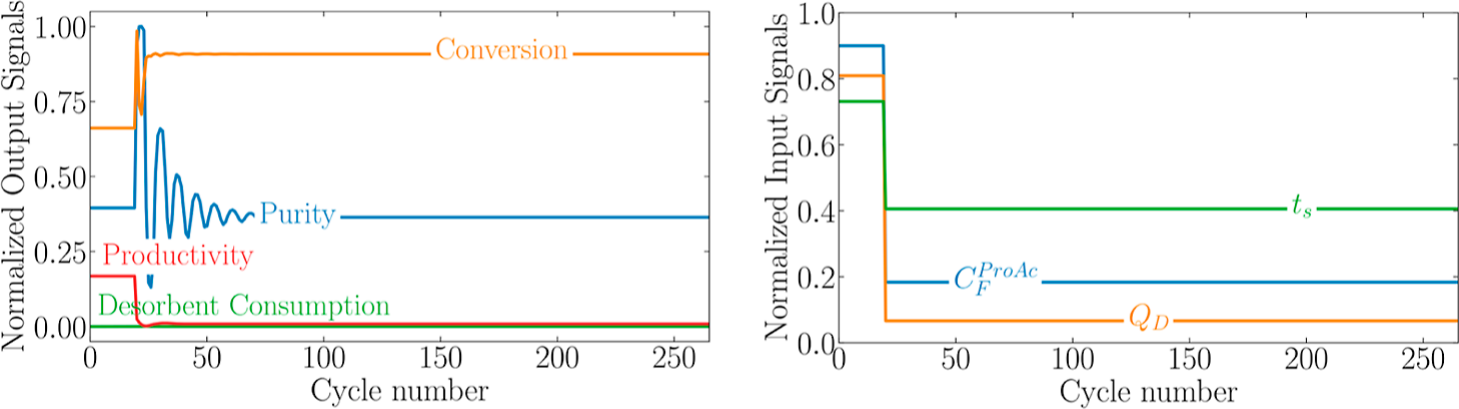
Input and output signals during experiment 13.

**Figure 5:**
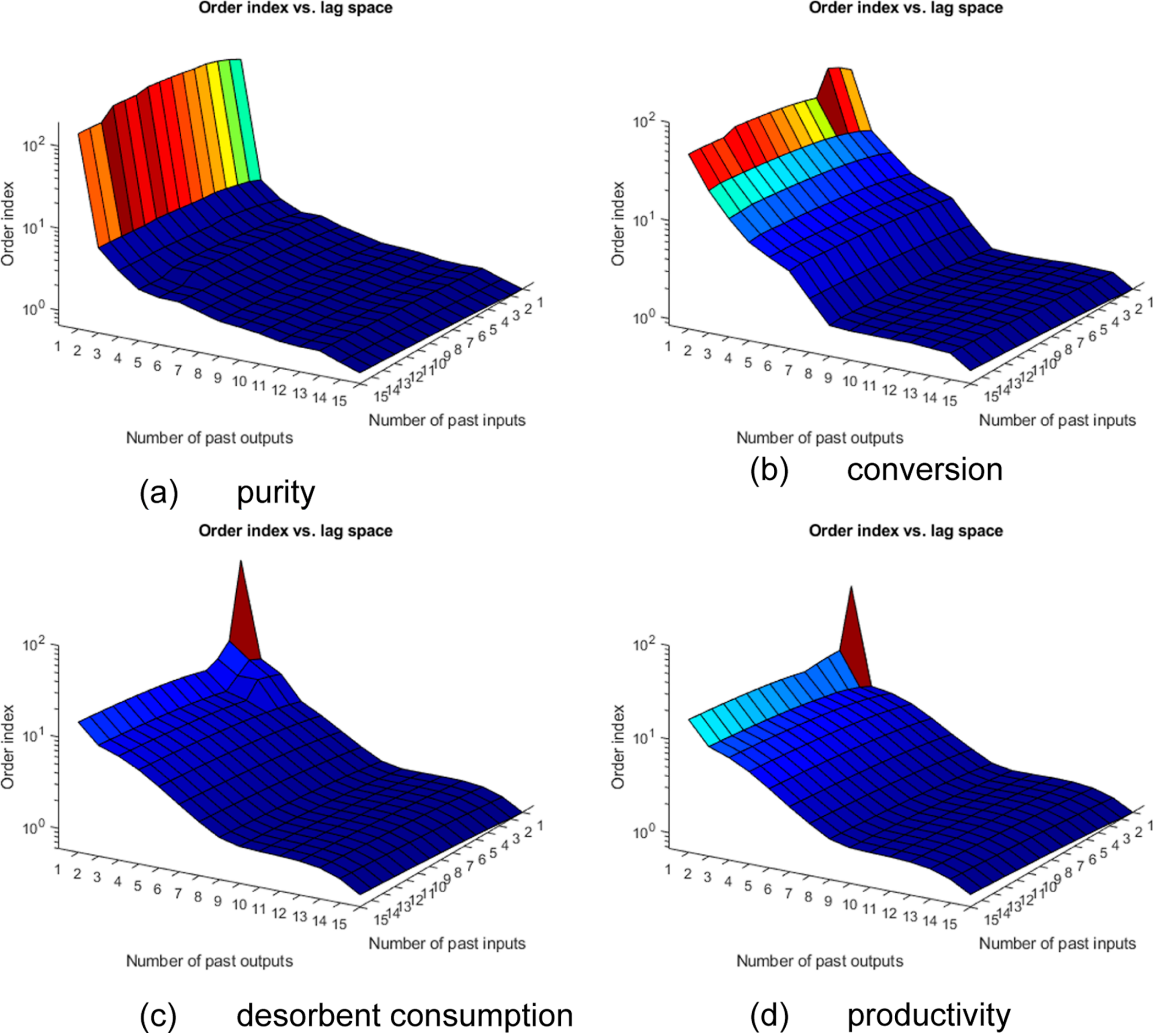
Lipschitz coefficients results for all performance indicators—(a) purity, (b) conversion, (c) desorbent consumption, and (d) productivity.

In Figure 3, ϵ represents the distance between the optimum analytical point and the optimum point found by the PSO. This concept allows us to determine the normalized squared error (*e*_*i*_^2^) concerning the variance (*V*_*i*_), according to the following equation
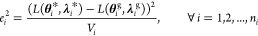
4where *n*_*i*_ is the number of decision variables of the PSO algorithm.

The error can be defined for all instances, *n*_*k*_, obtained in the optimization process, as follows
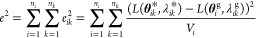
5the variance  between the global optimum and the total number of points considered in optimization  is defined as
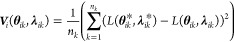
6combining 5 and 6
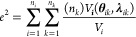
7at this point, a chi-squared distribution for *e*^2^, χ^2^, is assumed. This probability distribution has  degrees of freedom, where *n*_*k*_ is the total number of iterations developed by the PSO and *n*_y_ is the total number of objective functions considered in the optimization.
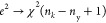
8

The error is calculated between the global analytical optimum and the remaining points belonging to the PSO population, represented in Figure 3, by *h*. The mathematical expression for *h* is defined as
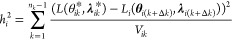
9extending the determination of error *h* to all points in each evaluation of the objective function about the analytic global optimum, we have the following normalized expression
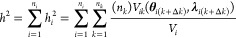
10where the variance *V*_*ij*_ can be defined by
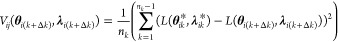
11therefore, the assumed chi-square distribution premise for *h*^2^ was also assumed for *e*^2^, whose degrees of freedom are expressed by , as follows
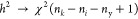
12thus, differences between the errors presented can be expressed by
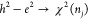
13these distributions are independent; it becomes a Fisher–Snedecor distribution as
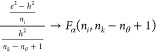
14where α is the confidence level of the Fisher–Snedecor test.

Finally, a Taylor series expansion can be derived around the optimal point to express the objective function
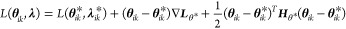
15where  is the gradient vector and  is the Hessian matrix of the objective function, which is correlated with the covariance matrix optimal points as
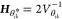
16replacing eq 14 in 13, one obtains
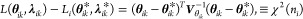
17simplifying eq 12, it is possible to rewrite it by equaling eq 15, obtaining
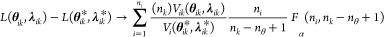
18assuming that *V*_*ij*_ is a good approximation for *V*, the optimal region meets the following test
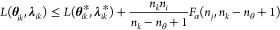
19

## 3 Results and Discussion

### 3.1 Identification

A set of 800 steps with ranges shown in Table 1 were created with LHS. Note that each step persisted for 250 time steps (prior estimated system settling time), leading to a total of 250.000 points. Three variables could be subject to step changes—concentration of propanoic acid in the feed stream (*C*_F_^ProAc^), desorbent flow rate (*Q*_D_), and switch time (*t*_s_), and four performance indicators outputs were collected—time-averaged conversion, productivity, desorbent consumption, and raffinate purity.

**Figure 6:**
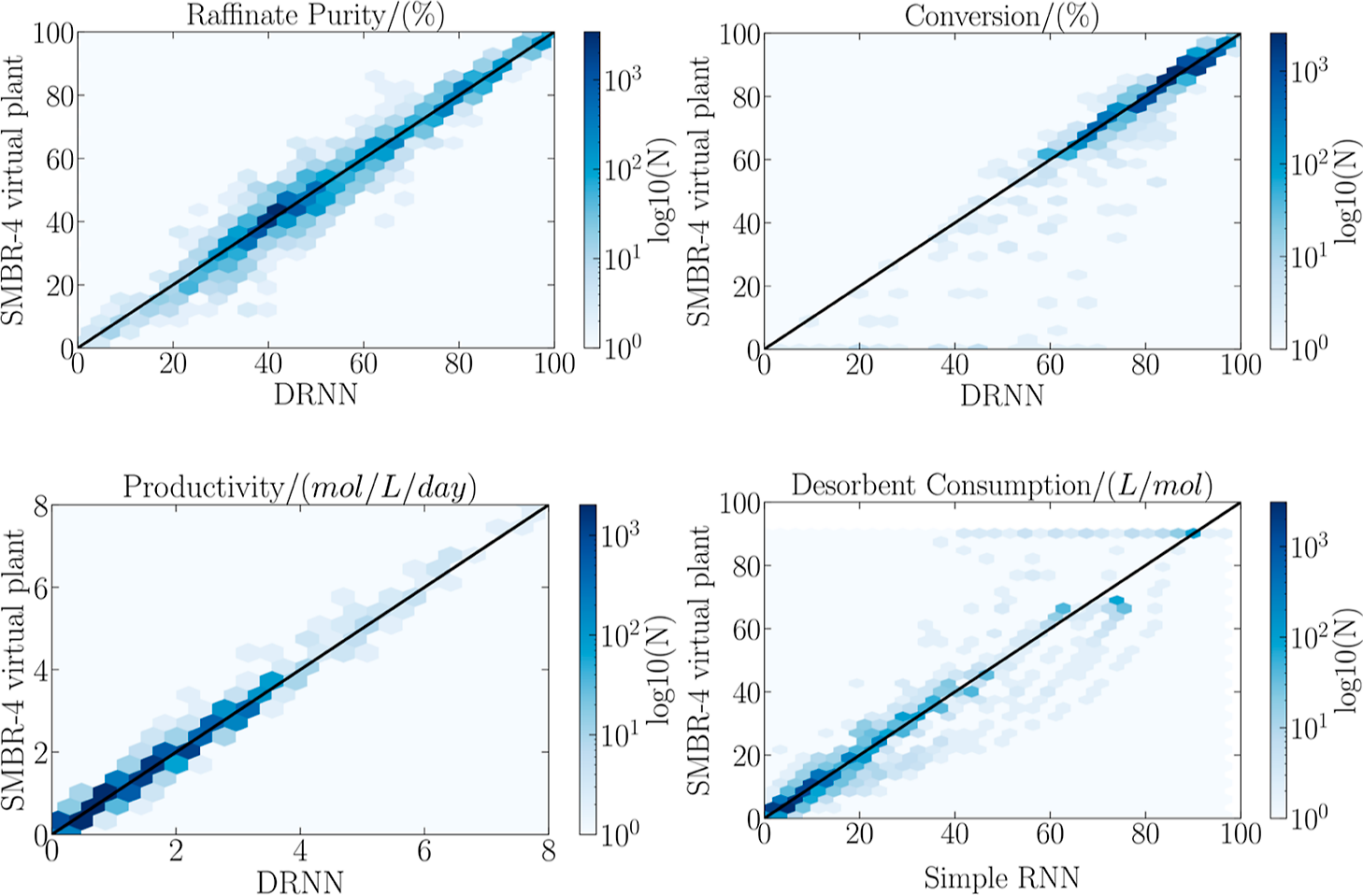
Hexabin parity plots for all performance indicators on the test set for the DRNN.

**Figure 7:**
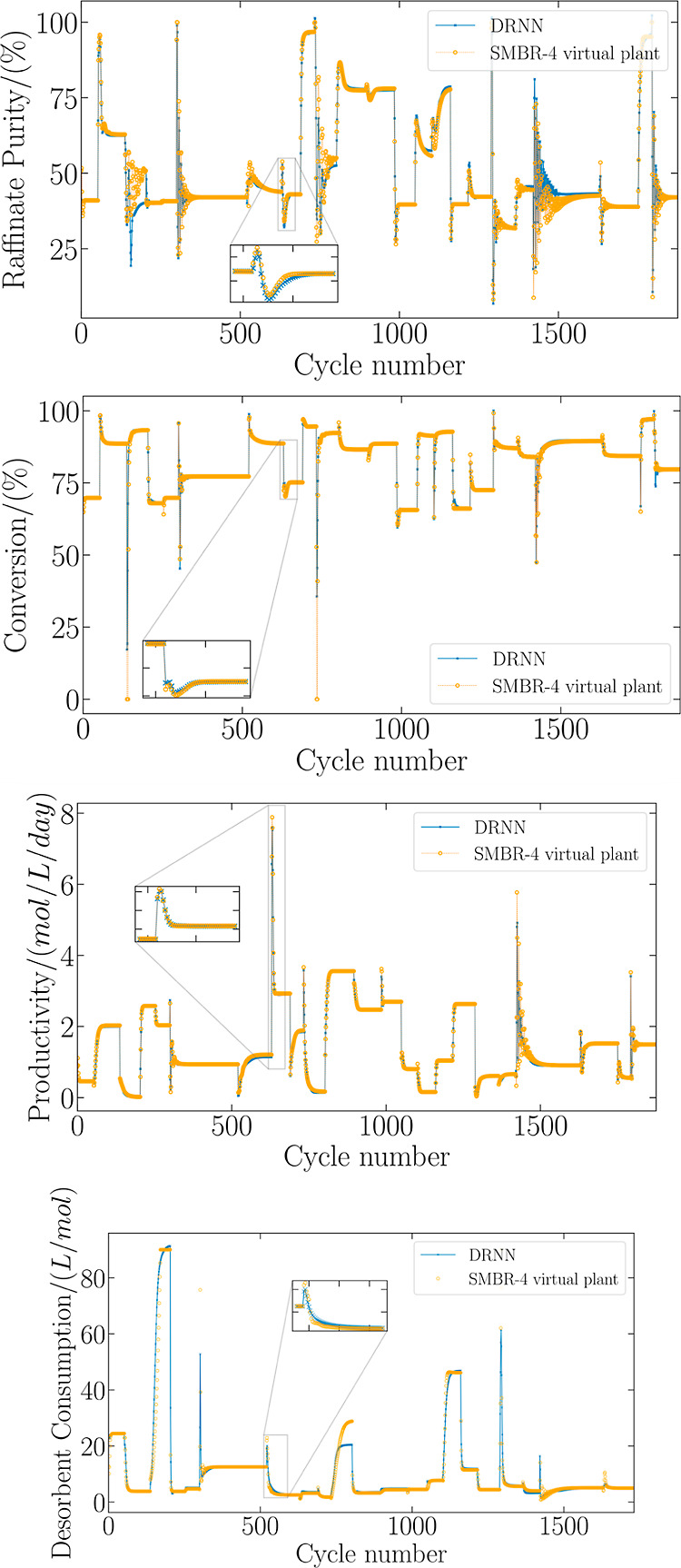
Comparison of DRNN predictions with a virtual SMBR-4 plant in the test set.

**Figure 8:**
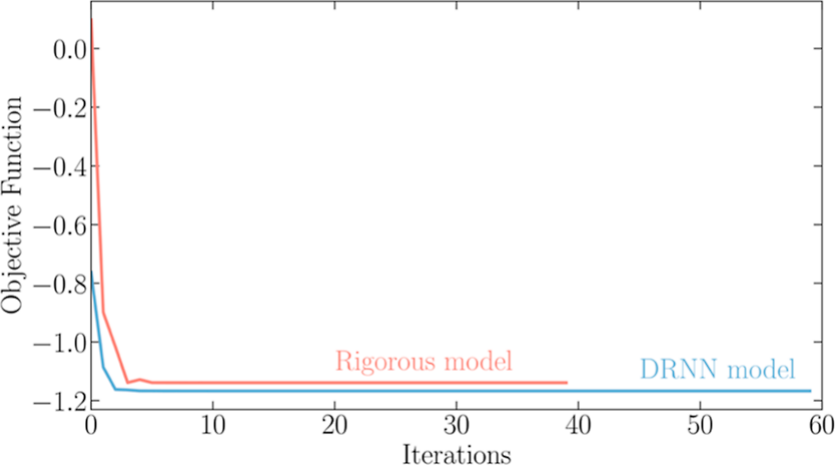
Evolution of swarm global best for 95% raffinate purity requirement.

The remaining input variables and parameters necessary to simulate the SMBR-4 model were taken from, namely, feed stream flow rate (*Q*_F_), recycling stream flow rate (*Q*_IV_), and extract stream flow rates (*Q*_X_), whose values are 1.400 × 10^–4^, 1.2135 × 10^–4^, and 9.000 × 10^–4^ (in dm^3^/min), respectively. Table 1 shows the ranges of the designed inputs as well as the outputs. The input space ranges were selected according to the physical limits of a lab-scale SMBR unit and made large enough to collect a considerable amount of information. It is worth remarking that the concentration of the limiting reactant cannot surpass its molar volume at working temperature, and it was the upper limit value for *C*_F_^ProAc^.

As described in 1.2, the original data set was partitioned into “experiments”. Figure 3 shows a sample of a single experiment described for both input and output signals. Note that all variables are already rescaled. It is possible to note that the performance indicators have very distinct dynamic behavior, which justifies the MISO approach. 680 patterns (85% of first data set) similar to the one presented in Figure 3 were used for training and the remaining ones were used for hyperparameter tuning. A separate data set was generated and used for testing.

As described, the Lipschitz coefficient method was used to determine the order, that is, assess long-term dependencies of the system encoded in *n*_a_ and *n*_b_ parameters. These numbers were determined using a graphical approach. This analysis was run for each performance indicator as it deals with a MISO case. The orders are defined as the pair (*n*_a_, *n*_b_) at which the order index stops changing significantly as the order increases. From Figure 3, it can be seen that the purity, conversion, desorbent consumption, and productivity orders are (4,1), (8,1), (9,1), and (9,1), respectively.

In order to find a combination of hyperparameters that result in the minimum mean squared error of the validation set, HYPERBAND parameter maximum epochs were set to 200. The factor (proportion of discarded configurations) was set to 5. The HYPERBAND method was used with early-stopping regularization to prevent overfitting. Additionally, the proposed optimality assessment is a robust method for demonstrating generalization capacity.

Table 2 shows the search space of the hyperparameters for the RNN.

Table 3 shows the best found hyperparameters for each performance indicator of the RNN. It can be seen that for some performance indicators, the best architectures involves stacked LSTM and GRU cells. A similar pattern is observed for the activation function.

These architectures achieve satisfactory performance for all performance indicators: 0.0243 for productivity, 0.209 for conversion, and 0.710 for purity (considering mean absolute error for the whole validation set). Figure 5 shows hexabin parity plots for all performance indicators for the DRNN in the test set. On the *x*-axis are the DRNN predictions, and on the *y*-axis are the test data. The dynamic data and predictions are also presented in Figure 6 for the test set.

One important aspect to consider in black-box models is the accuracy/computational time trade-off. While the gPROMs simulator takes about 566 s to run the response for one single step perturbation, the RNN surrogate takes about 150 ×10^–3^ seconds (both running in an Intel i7-7500U—2.7 GHz CPU). As is demonstrated in the next sections, the simulation time difference becomes essential for optimization.

### 3.2 Optimization for 95% Raffinate Purity Requirement

The PSO algorithm was used to solve the optimization problem described in eq 1 with both the rigorous and DRNN models. The decision vector is x = [, , ], where the variables are the scaled switching time, desorbent flow rate, and concentration of propanoic acid in the feed stream, respectively, according to Table 1. They are the same ones used to train the DRNN. Thus, the intervals were large enough to cover several possible operating conditions. However, expanding these intervals beyond the input space where the DRNN was trained can lead to inaccurate calculations of the objective function. It is a drawback of all surrogate models; that is, they can only be made accurate in the identification region. However, this work generated data in a broad region using LHS based on the constraints of a real lab-scale plant. Therefore, it ensures that the identified models will represent the system under evaluation well.

*w*_conv_ = −4 × 10^–3^, *w*_ec_ = 3 × 10^–1^, *w*_prod_ = −3 × 10^–1^, and *w*_pur_ = 50 were chosen as the set of weights in the objective function, and Pur_min_ was set to 95%. The weights reflect the objective from a process point of view—a negative value means that increasing the corresponding performance indicator magnitude will favor the objective function minimization and vice versa. Since the variables were rescaled, the upper and lower limits are 0 and 1, respectively.

When the SMBR-4 rigorous model was used in the optimization, 50 particles and 40 iterations were set. When the DRNN was used, 400 particles and 400 iterations were set. As mentioned in Section 2.1, the rigorous model is very costly to simulate. The number of particles and iterations is limited to the available time to perform the optimization. This specific case took 56 h with the rigorous model and 0.75 h with the DRNN.

Figure 4 shows the swarm minimum values’ (global best) evolution over the iterations. Even though the optimization of the DRNN was carried out up to 400 iterations, only 60 are shown. It can be seen that the convergence of the optimization with the DRNN model is quicker. Still, both objective functions stop decreasing before the 10th iteration.

Table 4 shows the decision variables at the optimal point for both rigorous and DRNN models. It can be seen that the decision vectors at the optimal point are close to each other, and the highest discrepancy is found for the desorbent flow rate component (*Q*_D_).

Table 5 shows the values of performance indicators that compose the objective function at the optimal point for each model and are evaluated using the same model; that is, DRNN optimal is evaluated with the DRNN model and rigorous optimal is evaluated with the rigorous model. It can be seen that the output values are close to each other. However, using single points to compare the optimization results of the two models can lead to misleading conclusions. One of the advantages of PSO is that it is a population-based algorithm which allows the building of the feasible operating region (FOR).

The DRNN optimal point could be run in the rigorous model, leading to an important perspective about the surrogate model optimal point robustness. However, the operating region characterization is a more complete and rigorous way of analyzing optimality. Furthermore, the proposed strategy is meant to demonstrate that the ANN result is equivalent to the rigorous model results. If this demonstration is done, it is proven that the optimizations are equivalent.

Figures 5 and 6 depict a 3D representation of swarm particles across iterations that meet the minimum purity requirement for both the rigorous model and DRNN, respectively. The *x-* and *y*-axis represent the scaled decision variables, and the *z*-axis is the objective function. The markers are colored according to the objective function value with a legend indicated in the color bar. In Figures 5b, 6b, 5c, and 6c, it can be seen that most of the swarm positions lie in a line where the concentration of the limiting reactant is close to the scaled upper limit 1. This value is a physical limit since the concentration of the component in the feed stream cannot surpass its molar density at a given temperature and pressure. Propanol feed concentration was calculated using its molar density for each value of propanoic acid concentration; that is, it is linearly correlated with *C*_F_^ProAc^ and removed from the inputs to avoid input space collinearity.

#### 3.2.1 Feasible Operating Region

The points obtained from the optimization with a rigorous model and the surrogate DRNN model are used to build their corresponding confidence regions. A more reliable way to verify if the two models lead to the same optimal point than simply comparing the global best is by comparing the two confidence regions. Suppose a significant degree of superposition of the two regions is verified. In that case, the surrogate model can be reliably used to optimize searches within the domain it was identified.

**Figure 9:**
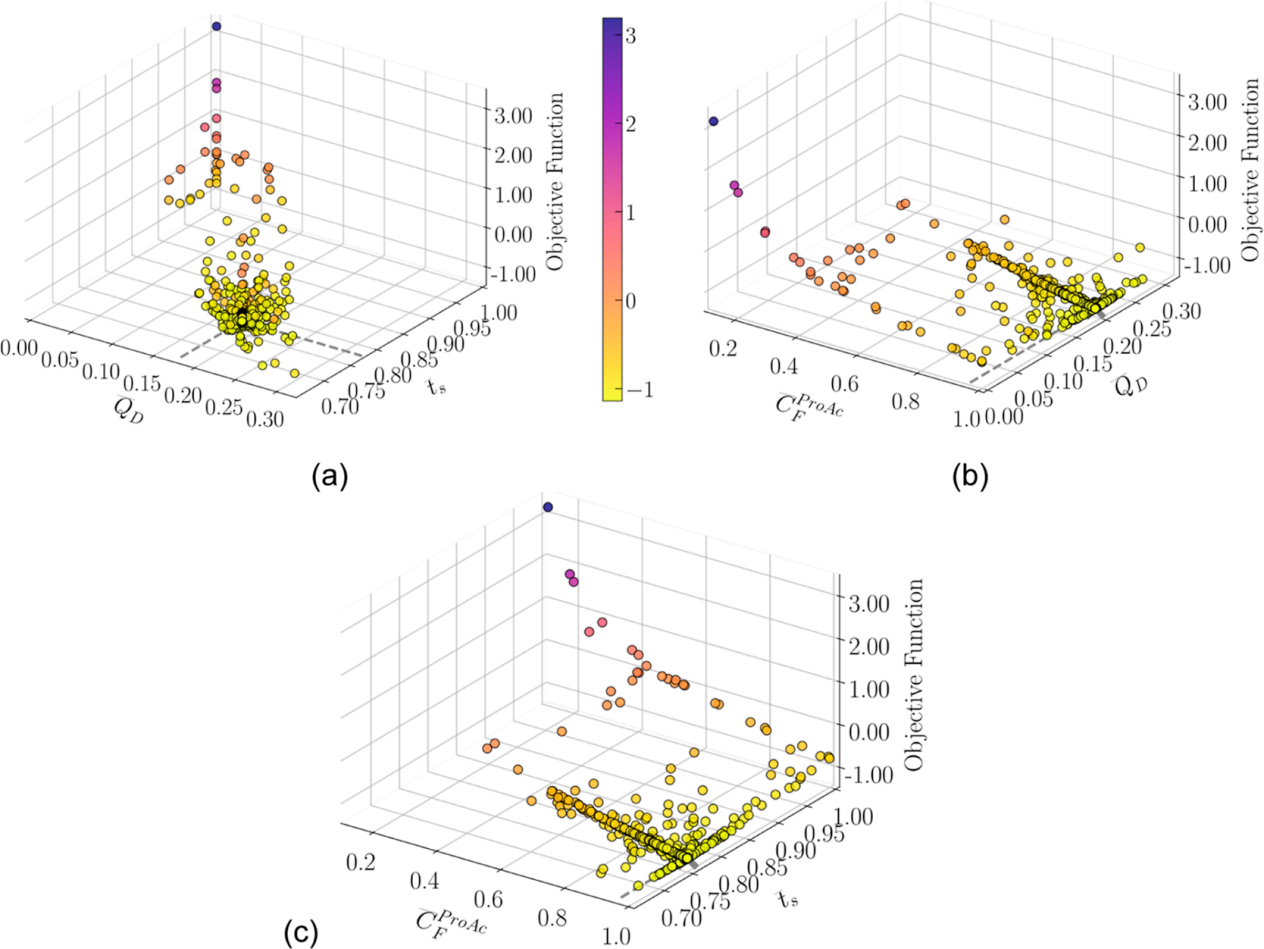
Swarm position and objective function for the rigorous model. (a) Desorbent flow rate vs switching time view, (b) desorbent flow rate vs limiting reactant feed concentration view, and (c) switching time vs limiting reactant feed concentration view.

**Figure 10:**
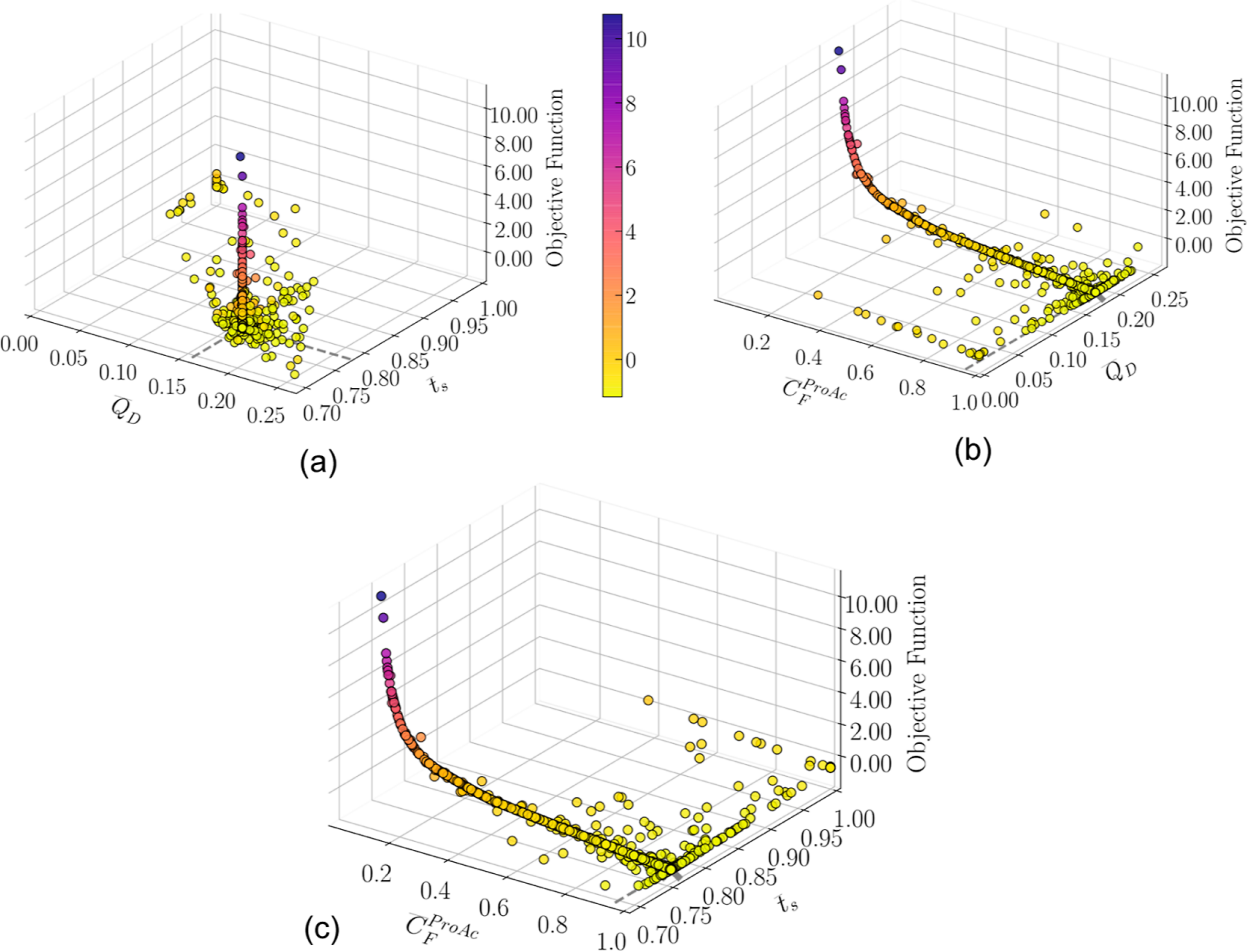
Swarm position and objective function for the DRNN model. (a) Desorbent flow rate vs switching time view, (b) desorbent flow rate vs limiting reactant feed concentration view, and (c) switching time vs limiting reactant feed concentration view.

**Figure 11:**
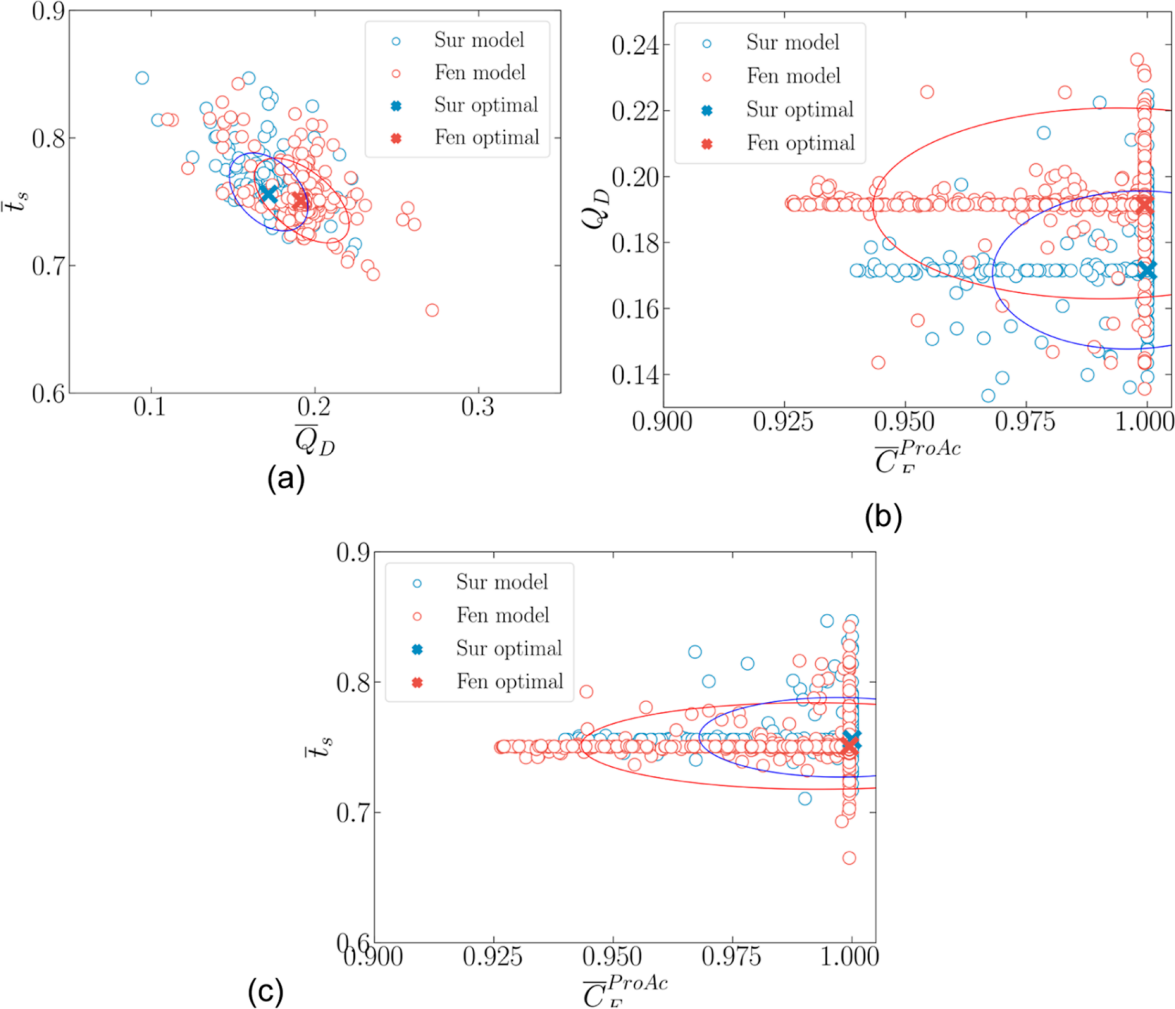
Scatter plot of points for 99.5% confidence level for rigorous (Fen) and surrogate (Sur) model (DRNN). (a) Desorbent flow rate vs switching time view, (b) limiting reactant feed concentration vs desorbent flow rate view, and (c) limiting reactant feed concentration vs switching time view.

Figure 7 shows a pair-wise scatter plot of the decision variables that passed a 99.5% test. It also includes the 99.7% probability ellipse (3 standard deviations) fitted using three bivariate Gaussian density distributions (one for each pair of inputs). In Figure 7a,b, it can be seen that most of the points lie in two lines of either constant  or  and , respectively, indicating that the Gaussian density may not be the ideal probability density function to fit. In Figure 7c, the Gaussian density seems to fit well in both clusters. Comparing the rigorous model with the DRNN, it can be seen that the points show a similar distribution pattern around their optimal points, and the ellipses have a significant degree of superposition. Moreover, it is possible to observe that the desorbent flow rate and the switching time have an inverse correlation, that is, less desorbent can be used, and similar performance can be achieved as soon as the switching time is increased (Figures 8–11).

## 4 Conclusions

The SMBR is challenging to optimize through first-principles models. In this sense, previous works in the literature concerning surrogates for its simpler version, the SMB, and other cyclic adsorptive processes indicate that using surrogate models may offer several benefits for SMBR optimization. However, there are no works published in the literature proposing surrogate models for the SMBR to our knowledge. Moreover, when surrogate models are used for optimization, optimality issues are barely considered; that is, only the optimal point found is tested in the rigorous model and used as the performance metric. It may lead to misleading conclusions about the model quality for optimization. In this setting, the present work shows that the proposed framework for SMBR units’ optimization based on DNN models and PSO optimizer with optimality evaluation provides a robust tool for SMBR surrogate identification and addresses optimality issues through the process feasible operating region.

A single objective function optimization problem was formulated by weighting the SMBR performance metrics. With this setting, a 448× speed up in the optimization time was observed when using the surrogate model compared to the rigorous SMBR model, with a significant overlap of the FOR regions for all decision variables. It is worth mentioning that this speed up is calculated to compare run times for online applications where both models are already identified and the goal is obtaining the optimal solution. Rigorous real-time optimization for such a system would be computationally infeasible due to the associated computational effort. The speed up is calculated by the ratio between mechanistic model simulation (1 dynamic response) elapsed time and surrogate model simulation elapsed time for the same response and in the same computer. Even sacrificing physics, the surrogate model is feasible for real-time usage, where the fully mechanistic model, despite being run in gPROMs with a top desktop processor, fails to achieve it. Moreover, the rigorous model used is not easily parallelizable and would not benefit significantly from a high-performance computing environment as it is a stiff system of partial differential equations, which does not have a steady state but a cyclic one.

This work built the surrogate model from noise-free data, that is, from rigorous numerical simulations of the real SMBR model. In future works, one may consider introducing computer-generated noise to simulated data and evaluating its effects on the model and the process optimization. However, the results obtained prove that the surrogate model represents the system behavior with precision. This is clearly seen by the optimality analysis. Finally, an optimal FOR was obtained. The SMBR can be operated with high conversion while providing high purity ProPro using the obtained FOR.

## Appendix

The model equations and parameters are displayed in Tables A1, A2, and A3.

**Table A1:** SMBR—4 Model Equations

Material balance in volume elements of column *k* for component *i* and velocity variation equation:
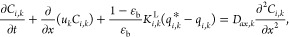
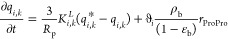
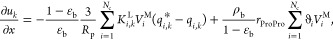
*N*_C_ is the total number of components, *x* is the axial position, *t* is the time, ϑ is the stoichiometric coefficient, *r*_ProPro_ is the reaction rate, ρ_b_ is the bulk density, *D*_ax_ is the axial dispersion coefficient, q* is the adsorbed molar concentration in equilibrium with *C* (bulk phase molar concentration), *q* is the average adsorbed molar concentration, *u* is the liquid interstitial velocity, *R*_P_ is the mean particle radius, ε_b_ is the bed porosity, *K*^L^ is the external mass transfer coefficient, and *V*^M^ is the molar volume.
Initial and boundary conditions for each component *i* and column *k*:
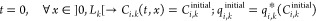
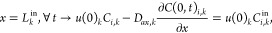
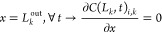
*L*_k_^in^ and *L*_k_^out^ are the inlet and outlet positions in section *k*, respectively

Global and component balances in nodes:		
desorbent node: 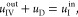	feed node: 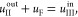	extract node: 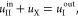
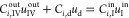	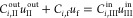	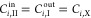
raffinate node: 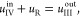	intermediate nodes: 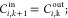	
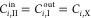	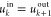	

Kinetic and equilibrium equations:
*isotherm model:*
*Q*_i_^max^ is the maximum adsorption capacity, *K*_i_ is the adsorption constant
*Chemical reaction equation:*
; ProAc is propanoic acid, POH is 1-propanol, H_2_O is water, and ProPro is *n*-propyl-propionate
*Reaction rate model:*
, *a* is the activity coefficient

*Mass transfer coefficient correlations:*		
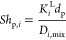	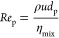	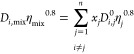
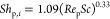	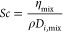	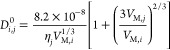
*Sh*_p_, *Re*_P_, and *Sc* are the Sherwood, Reynolds, and Schmidt numbers, respectively, *d*_p_ is the mean particle diameter, *D*_*i*,mix_ is the mixture diffusion coefficient, ρ is the fluid density, η_mix_ is the mixture viscosity, and *D*_*i*.*j*_^0^ is the infinity dilution diffusion coefficient.		

Performance indicators:		
conversion: 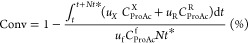	purity: 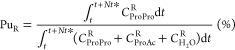	productivity: 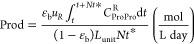
desorbent consumption: 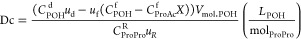		

**Table A2:** Langmuir Multicomponent Competitive Model Parameters

component	*K*/(L/mol)	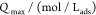
POH	11.66	9.13
ProAc	9.04	10.06
ProPro	5.08	5.11
H_2_O	2.35	43.07

**Table A3:** Parameters of Columns in TMBR

parameter	value
ε_b_	4 × 10^–1^
*P*_e_	166
*R*_p_/(μm)	122.75
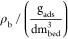	390
*L*/(dm)	4.6
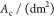	4.15 × 10^–2^
*T*/(K)	313
